# A Methodical Framework Utilizing Transforms and Biomimetic Intelligence-Based Optimization with Machine Learning for Speech Emotion Recognition

**DOI:** 10.3390/biomimetics9090513

**Published:** 2024-08-26

**Authors:** Sunil Kumar Prabhakar, Dong-Ok Won

**Affiliations:** Department of Artificial Intelligence Convergence, Chuncheon 24252, Republic of Korea; sunilprabhakar22@gmail.com

**Keywords:** SER, transforms, feature selection, classification, ELM

## Abstract

Speech emotion recognition (SER) tasks are conducted to extract emotional features from speech signals. The characteristic parameters are analyzed, and the speech emotional states are judged. At present, SER is an important aspect of artificial psychology and artificial intelligence, as it is widely implemented in many applications in the human–computer interface, medical, and entertainment fields. In this work, six transforms, namely, the synchrosqueezing transform, fractional Stockwell transform (FST), K-sine transform-dependent integrated system (KSTDIS), flexible analytic wavelet transform (FAWT), chirplet transform, and superlet transform, are initially applied to speech emotion signals. Once the transforms are applied and the features are extracted, the essential features are selected using three techniques: the Overlapping Information Feature Selection (OIFS) technique followed by two biomimetic intelligence-based optimization techniques, namely, Harris Hawks Optimization (HHO) and the Chameleon Swarm Algorithm (CSA). The selected features are then classified with the help of ten basic machine learning classifiers, with special emphasis given to the extreme learning machine (ELM) and twin extreme learning machine (TELM) classifiers. An experiment is conducted on four publicly available datasets, namely, EMOVO, RAVDESS, SAVEE, and Berlin Emo-DB. The best results are obtained as follows: the Chirplet + CSA + TELM combination obtains a classification accuracy of 80.63% on the EMOVO dataset, the FAWT + HHO + TELM combination obtains a classification accuracy of 85.76% on the RAVDESS dataset, the Chirplet + OIFS + TELM combination obtains a classification accuracy of 83.94% on the SAVEE dataset, and, finally, the KSTDIS + CSA + TELM combination obtains a classification accuracy of 89.77% on the Berlin Emo-DB dataset.

## 1. Introduction

Emotions have been found to have a significant influence on the psychological and physical well-being of human beings [1]. The sharing of emotions by patients and how well they are received by therapists’ aid in the assessment of good treatment options. A huge amount of data is processed by therapists over a long period of time, which is quite a tumultuous task [2]. Thus, if a speech-based emotion training process is made feasible, it would be highly beneficial to therapists, as it would save a lot of time and energy. For instance, voice samples portraying different emotions, such as sadness, surprise, joy, anger, and neutral mode, could be considered and used to train a neural network to allow for their recognition [3]. Without the need for intrusive technology, spoken audio signals can be examined so that emotional information can be obtained. Using various applications, emotions have been grouped into text and audio based on how they evolve with time [4]. For human communication, emotion serves as an utmost important skill so that interpersonal connections can be managed well. With the help of emotional inputs, multiple cognitive computing tasks are achieved, and they sometimes greatly aid in perception and rational thinking. Some versatile examples of emotion classification and recognition include banking, computer games, video and audio monitoring, and psychiatric diagnosis [5]. Emotional speech recognition can be utilized for entertainment purposes, clinical investigations, online learning, and corporate applications. When voice signals are combined with emotions, it can create a holistic method called emotion recognition from speech. This method was chosen in this research as it has a negligible cost and is more appealing and useful than processing other biomedical signals [6,7]. In short, SER is widely used to trace and identify emotions in human speech signals. This technique has received a lot of attention in the last decade, as it bridges the gap and serves as an important link in human–computer interfaces [8]. SER has been applied for drowsiness detection, emotional state assessment, sleep stage scoring, medical diagnosis, etc. In the past few years, various SER studies have been conducted, primarily based on acoustic features [9]. They include assessments of voice quality, spectral features, prosodic features, etc., implemented for emotion recognition. Earlier studies used either one type of acoustic feature to identify emotions or multiple features to identify emotions, supported by classification with traditional machine learning or deep learning classifiers [10]. Some of the common works carried out in the past few years are discussed below. In this work, experiments were conducted on four publicly available datasets, namely, EMOVO [11], RAVDESS [12], SAVEE [13], and Berlin Emo-DB [14], and results previously reported on these datasets are discussed specifically for performance comparison analyses.

Regarding the EMOVO dataset, the following techniques and results have been reported: speaker awareness for SER was analyzed, obtaining a classification accuracy of 68.50% [15]; automatic feature selection techniques were applied for emotion recognition in low-resource settings, obtaining a classification accuracy of 41% [16]; novel feature selection techniques were applied for entire recognition, obtaining a classification accuracy of 60.40% [17]; and the transfer learning concept was applied to enhance SER where the knowledge gained through one task or dataset is utilized to improve the model performance on another related task or different dataset, obtaining a classification accuracy of 76.22% [18].

Regarding the RAVDESS database, the following techniques and results have been reported: machine learning techniques were implemented, obtaining a classification accuracy of 80.21% [19]; convolutional neural networks (CNNs) were implemented, obtaining a classification accuracy of 79.50% [20]; multimodal SER using CNN was implemented, obtaining a classification accuracy of 78.20% [21]; spiking neural networks were implemented, obtaining a classification accuracy of 83.60% [22]; the capsule routing technique was implemented, obtaining a classification accuracy of 77.02% [23]; bagged SVM was implemented, obtaining a classification accuracy of 75.69% [24]; spectrogram-based multi-task audio classification was carried out, obtaining a classification accuracy of 64.48% [25]; frequency cepstral coefficients with neural networks were applied, obtaining a classification accuracy of 79.80% [26]; and continuous wavelet transform (CWT) was applied, obtaining a classification accuracy of 60.10% [27].

Regarding the SAVEE dataset, the following techniques and results have been reported: a hierarchical classifier was used for SER, obtaining a classification accuracy of 83.78% [28]; joint deep cross-domain transfer learning was used, obtaining a classification accuracy of 69.00% [29]; the concept of negative emotion recognition was used, obtaining a classification accuracy of 65.83% [30]; 3D-CNN-based SER was carried out using K-means clustering and spectrograms, obtaining a classification accuracy of 81.05% [31]; recurrence dynamics were integrated for SER, obtaining a classification accuracy of 80.20% [32]; a comparison of cepstral features for SER was carried out, obtaining a classification accuracy of 78.60% [33]; and hybrid Particle Swarm Optimization (PSO)-based biogeography optimization was carried out for emotion and stress recognition, obtaining a classification accuracy of 78.44% [34].

Regarding the Berlin Emo-DB database, the following techniques and results have been reported: a two-layer fuzzy multiple random forest concept obtained a classification accuracy of 87.85% [35], a modified quantum-behaved PSO obtained a classification accuracy of 82.82% [36], a wavelet packet analysis obtained a classification accuracy of 79.50% [37], multi-time-scale convolution obtained a classification accuracy of 70.97% [38], voting mechanisms obtained a classification accuracy of 64.52% [39], the stacked generalization method obtained a classification accuracy of 82.45% [40], and the divide-and-conquer-based ensemble technique obtained a classification accuracy of 82.00% [41].

**Figure 1:**
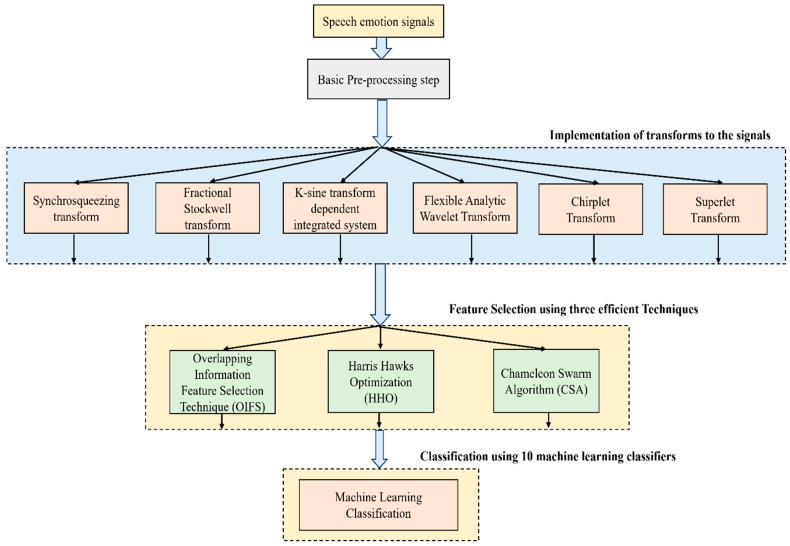
Simplified illustration of this work.

The main contributions of this work are as follows:

(a) After the basic pre-processing of the signals is carried out using an Independent Component Analysis (ICA), the pre-processed signals are subjected to six transforms, and then features are extracted.
(b) The extracted features are selected using three efficient techniques, namely, OIFS, HHO, and CSA, and they are finally fed into machine learning classifiers.
(c) While the techniques may already be established, the combinations with which the workflow proceeds are completely novel and interesting, as no previous works have reported the combination of transforms with optimization techniques, followed by classification with machine learning classifiers. A simplified version of a block diagram for a holistic understanding is presented in Figure 1.

## 2. Implementation of Transforms

The following six important transforms were applied to pre-processed speech emotion signals: the synchrosqueezing transform, FST, KSTDIS, FAWT, chirplet transform, and superlet transform. Some of the advantages considered when choosing these transforms are that they should be computationally very fast and these transforms should be able to explore the fine and innate details of the signal clearly. Some of the transforms can also provide a good and simultaneous localization in both time and frequency domain. The implementation aspects should also be easy so that it could ease the burden of the researchers to a great extent. The implementation of these transforms is described below.

### 2.1. Synchrosqueezing Transform

The synchrosqueezing transform is highly dependent on the continuous wavelet transform (CWT) [42]. This transform is highly useful for obtaining the localized time–frequency specifications of non-stationary signals and the oscillation components of a signal. The basic form of the signal q(t) is indicated as follows:

(1) $$q(t)=\sum_{n=1}^{N}q_{n}(t)+e(t)$$

where every component in $q_{n}(t)=M_{n}(t)\cos\left(\phi_{n}(t)\right)$ represents an oscillation function with time-varying frequency and amplitude, and the noise is specified by e(t). For every component in n=1,…,N, the intention is to obtain the amplitude $M_{n}(t)$ and the instantaneous phase $\phi_{n}(t)$. Hilbert transforms are applied to the original $q_{n}(t)$ component, and the formation of the analytic signal $a_{n}(t)$ is obtained as follows:

(2) $$a_{n}(t)=M_{n}(t)e^{j\phi_{n}(t)}=q_{n}(t)+iH\left\{q_{n}(t)\right\}$$

For the multicomponent signal, $a_{n}(t)$ is extended by means of a vector construction to every analytic signal of the channel; therefore, a multivariate analytic signal is obtained as follows:

(3) $$a_{n}(t)=\left[\begin{array}{c} \begin{array}{c} M_{1}(t)e^{j\Phi_{1}(t)}\\ M_{2}(t)e^{j\Phi_{2}(t)}\\ M_{3}(t)e^{j\Phi_{3}(t)}\\ M_{4}(t)e^{j\Phi_{4}(t)}\\ :\\ : \end{array}\\ :\\ M_{N}(t)e^{j\Phi_{N}(t)} \end{array}\right]$$

Every speech emotion is represented as a monocomponent signal, as shown in Equation (3). For the multicomponent signal q(t), the highly localized time–frequency indication is represented by the SST algorithm utilizing the instantaneous amplitude. By utilizing the CWT, the frequency information is extracted from the analytic signal. The oscillatory component of a particular signal is imbibed by the CWT algorithm via the use of time–frequency filters termed wavelets. The finite oscillation function is represented by the mother wavelet ψ(t), and it is usually convolved with the signal q(t). The computation of the CWT of $q_{n}(t)$ is carried out as follows:

(4) $$W\left(c,d\right)=\int c^{-\frac{1}{2}}\Psi \left(\frac{t-d}{c}\right)q_{n}(t)dt$$

Here, the wavelet coefficients for every scale–time pair (c,d) are indicated by W(c,d). The bandpass filter output consists of only the wavelet coefficients. In the frequency domain, the bandpass filter is scrolled by the scale factor ‘c’ so that the bandwidth of the filter changes. The scale parameter c highly influences the frequency properties, while the time of interest is indicated by the shift parameter ‘d’. Therefore, at a particular frequency of $f_{s}$, the spreading of the energy of a sinusoidal wavelet transform occurs around a scale factor and is represented as cs=fψfs. Here, $f_{\psi }$ indicates the wavelet’s center frequency, and the energy of the original frequency $f_{s}$ is widely spread across $c_{s}$. The actual frequency $f_{r}$ and the estimated frequency in the relevant scales are similar. For every scale–time pair (c,d), the evaluation of the instantaneous frequency $f_{q}(c,d)$ is carried out as follows:

(5) $$f_{q}(c,d)=-iF\left(c,d\right)\frac{-1\partial F\left(c,d\right)}{\partial d}$$

The wavelet coefficients are transformed from the scale–time domain to the time–frequency domain using the SST technique. Every point (c,d) is transformed into $\left(d,f_{q}\left(c,d\right)\right)$. A scaling step is required, as c and d are discrete values. The computation of $\Delta c_{k}=c_{k-1}-c_{k}$ for any $c_{k}$, where $f_{q}(c,d)$, is carried out. When the domain transformations are carried out (from the scale–time domain to the time–frequency domain), $\left(d,c\right)\to \left(d,f_{inst}\left(c,d\right)\right)$, the computation of the SST coefficient $T\left(f_{l},d\right)$ is carried out at the center $f_{l}$ of the frequency range $\left(f_{l}-\frac{\Delta f}{2},f_{l}+\frac{\Delta f}{2}\right)$, with $\Delta f=f_{l}-f_{l-1}$. At a resolution of Δf, the frequency bias is indicated by $f_{l}$. The linear frequency scales are specified by Δf, and this acts as a constant value. The oscillations in the univariate mode are reconstructed using SST so that $q_{n}(t)=M_{n}(t)\cos\left(\phi_{n}(t)\right)$. The reconstructed signal q(d) and the synchrosqueezing coefficient are expressed as follows:

(6) T(fl,d)=∑ck:|fq(ck,d)−fl|≤Δf2F(ck,d)c−32Δck

(7) $$q\left(d\right)=ℜ\left[R_{\psi }^{-1}T\left(f_{l},d\right)\Delta f\right]$$

The time–frequency indication of the signal is represented by Equation (4) and is synchrosqueezed along with the frequency scale. In Equation (7), $R_{\psi }=\frac{1}{2}\int_{0}^{\infty }{\tilde{\psi }}^{*}\left(\xi \right)\frac{d\xi }{\xi }$ represents the normalization constant. For the mother wavelet ψ(t), $\hat{\psi }\left(\xi \right)$ represents the Fourier transform. To maintain a consistent indication in the time–frequency domain, the coefficient of the CWT is reallocated using the SST algorithm so that instantaneous frequencies are automatically generated. The multivariate synchrosqueezing transform is explained as follows: The expressive indication in the time–frequency domain is provided using the modulated oscillation model. A multivariate extension is presented to the synchrosqueezing transform so that the common oscillations pertaining to the multiple data channels are recognized. For multivariate data such as speech emotion data, the procedure is started by applying the SST to every channel. The synchrosqueezing coefficients are initially obtained, and then the multiple channels are assigned depending on the rules. From a collection of multivariate signals, a group of monocomponent signals are identified, and the separation of the time–frequency domain components into K frequency band components is carried out as ${\left\{f_{k}\right\}}_{k=1,\ldots ,K}$. Assessments of the instantaneous frequencies and amplitude are conducted. The computation of the multivariate instantaneous frequency and amplitude is carried out across the channels, and, ultimately, an assessment of the multivariate synchrosqueezing coefficient is conducted.

### 2.2. Fractional Stockwell Transform (FST)

Among the prevalent joint time–frequency analysis tools, one of the most famous techniques is the FST, as it helps to improve the time–frequency concentration to a greater extent [43]. Here, two widely used transforms are combined—the fractional Fourier transform (FrFT) and the Stockwell transform (ST). For the signal q(t), a $z^{th}$-order continuous and discrete FST is represented mathematically as follows:

(8) $$FST_{q}^{2}\left(\tau ,v_{\varphi }\right)=\int_{-\infty }^{\infty }q(t)h\left(\tau -t,v_{\varphi }\right)K_{z}\left(t,v_{\varphi }\right)dt$$

and

(9) $$FST_{q}^{z}(k,p)=\sum_{n=-N}^{N}q(n)h\left(k-n,p\right)K_{z}\left(n,p\right)$$

where the kernel function of the FrFT is expressed as $K_{z}(t,v_{\varphi })$, which is expressed as follows:

(10) $$K_{z}(t,v_{\varphi })=\{\begin{array}{c} \sqrt{\frac{1-j\cot\varphi }{2\pi }}\exp\left[j\left(\frac{t^{2}+v_{\varphi }^{2}}{2}\right)\cot\varphi -jv_{\varphi }t\csc\varphi \right],\varphi \neq n\pi \\ \delta (t-v_{\varphi })if\begin{array}{cc} & \varphi =2n\pi \end{array}\\ \delta (t-v_{\varphi })if\begin{array}{cc} & \varphi +\pi =2n\pi \end{array} \end{array}$$

The scalable Gaussian window function is expressed as $h\left(\tau -t,v_{\varphi }\right)$; it is dependent on time t and the fractional Fourier frequency $v_{\varphi }$, and it is represented as follows:

(11) $$h\left(t,v_{\varphi }\right)=\frac{\left|v_{\varphi }\csc\varphi \right|}{\sqrt{2\pi }s}\exp\left(\frac{-t^{2}{\left(v_{\varphi }\csc\varphi \right)}^{2r}}{2s^{2}}\right)$$

where φ=zπ2 and 0<|z|<2. There are two window adjustment blocks of parameters alongside the fractional order parameter (z), namely, r and s, which help to mitigate the window shape. The energy of the signal is localized using these parameters so that the time–frequency resolution is improved. The calculation of the FST is carried out as follows:

In the context of chirp basis functions, the Gaussian-windowed signals are decomposed with respect to the phase and time, and they are highly influenced by different $v_{\varphi }$ values. In the time–frequency plane, it is best to analyze the non-stationary signals, as they are more interpretable and robust. As it provides a good frequency and time resolution, the ST remains a very famous window-based technique among the joint time–frequency analysis tools. At lower frequencies, the frequency resolution is improved, and, at higher frequencies, a good time resolution is provided. Despite having such versatile features, it has also certain shortcomings. The time resolution is sometimes degraded, as the Gaussian window function is quite lengthy with tapering ends; therefore, the pertinent data present in the signal are suppressed. The window shape parameters in the ST cannot be adjusted, as they are highly dependent on the frequency. Therefore, to improve performance, these limitations must be overcome successfully, and, to this end, a fractional variation of the ST called the FST is used in this work. To eliminate noise, the fractional Fourier domain is quite effective when dealing with non-stationary signals. The noisy counterparts can be separated in the intermediate time–frequency domain using the fractional order of the transform, and a better resolution can be obtained using the ST. The extension of the ST in the fractional domain is aided as the signal localization is improved, thereby enhancing the flexibility of the entire spectrum of the signal. Thus, to process the noisy content of the signals, FST is utilized. A scalable fractional Gaussian window is utilized by this modified variant of the ST in the fractional domain with the help of the fractional parameter (z) and two window adjustment parameters r and s. A good improvement can be seen in the resolution when using these two window adjustment parameters and fractional parameter. The rotation angle can also be changed so that even multi-resolution spectrograms can be achieved, which are sometimes large, thereby improving the flexibility.

### 2.3. K-Sine Transform-Dependent Integrated System (KSTDIS)

A K-sine transform-dependent integrated system is described below to investigate its effectiveness [44]. The sinusoidal transformation exhibits a very powerful nonlinear dynamic behavior and it can be implemented to chaotic systems quite easily. With the help of the sine map, the chaos of a chaotic system can be enhanced as follows:

(12) $$v_{1}=y\sin\left(\pi Q\right),y\in \left(0,1\right)$$

The K-sine map is dependent on the sine map, and it is expressed as follows:

(13) $$v_{2}=\left|y\sin\left(k\pi q\right)\right|,y\in \left(0,1\right)$$

The chaos of the function is enhanced by $v_{2}$ with the mitigation of periodicity of the trigonometric function. If the value of k=1, then $v_{2}$ becomes equivalent to $v_{1}$. The chaotic property of $v_{2}$ is higher than that of $v_{1}$, and it is specified with the help of the Lyapunov exponent [45] and sample entropy [46]. The numerical features of adjacent trajectories are indicated by the Lyapunov exponent, and this aids in tracing the chaotic motion. To identify the chaos of a system, the LE is highly useful. For a variable chaotic system, $Q_{i+1}=F\left(Q_{i}\right)$, and its Lyapunov exponent is expressed as follows:

(14) $$\lambda_{F}=\underset{n\to \infty }{\lim}\frac{1}{n}\sum_{i=1}^{n-1}\ln\left|F^{\prime }\left(Q_{i}\right)\right|$$

The system progresses towards a chaotic state if $\lambda_{F}>0$, and such a mapping is termed a chaotic mapping. If the $\lambda_{F}$ value is large, then the chaotic behavior is quite complex in nature. To assess the time series complexity, sample entropy is highly useful when compared with approximate entropy. The time series complexity is assessed using sample entropy, and this is carried out by specifically measuring the probability of a normal pattern in the time sequences. The complexity will be higher if the probability of the generated new patterns is high. The complexity will be lower if the sample entropy value is small and the time series self-similarity value is high. If the sample entropy value is large, then the time series will be more complex. Based on $v_{2}$, this study proposes the use of KSTDIS, and its mathematical model is represented as follows:

(15) $$Q_{i+1}=\left|\sin k\pi \left(F_{1}\left(q_{i},y\right)+F_{2}\left(q_{i},1-y\right)\right)\right|$$

where $F_{1}\left(q_{i},r\right)$ and $F_{2}\left(q_{i},1-r\right)$ are two known 1D chaotic map seeds. The control parameters are k and y, k∈[1,∞],y∈[0,1], and the absolute value is denoted by |⋅|. The coupling operation is performed on $F_{1}\left(q_{i},y\right)$ and $F_{2}\left(q_{i},1-y\right)$. Then, sine transformation is performed on the coupling result with the help of $v_{2}$. The chaotic dynamics can be easily absorbed by the coupled operation. The merits of the nonlinear dynamic behavior of the sine transform are greatly taken care of, and, thus, the arbitrary selection of seed maps can be carried out so that novel chaotic maps larger in number can be generated. The effectiveness of the KSTDIS must be proven. Here, k is considered to have a value of 3, and the chosen seed chaos is a logistic map, a sine map, and a tent map.

Sine map:

(16) $$q_{i+1}=y\sin\left(\pi q_{i}\right)$$

Logistic map:

(17) $$q_{i+1}=4yq_{i}\left(1-q_{i}\right)$$

Tent map:

(18) $$q_{i+1}=\{\begin{array}{cc} 2yq_{i}, & q_{i}<0.5\\ 2y\left(1-q_{i}\right), & q_{i}\geq 0.5 \end{array}$$

Then, the control parameters for all the maps are the variables r and r∈[0,1]. The mathematical model for the 3-KSTDIS is expressed as follows:

(19) $$Q_{i+1}=\left|\sin\left(3\pi \left(F_{1}\left(q_{i},y\right)+F_{2}\left(q_{i},1-y\right)\right)\right)\right|k\in \left[1,+\infty \right),y\in \left[0,1\right]$$

Here, the three seed mappings are not combined, as the chosen value of k is only 3.

### 2.4. FAWT

To analyze speech emotion signals, the analytic wavelet transforms with flexible time–frequency covering is utilized [47]. The Hilbert transform pairs of atoms are utilized by this transform so that a higher flexibility is provided, thereby controlling the dilation factor, redundancy, and Quality Factor (*QF*). The signals can be easily analyzed by adjusting the parameters: the down sampling factor for the low-pass channel (l) and high-pass channel (h) and the up-sampling factor for the low-pass channel (u) and high-pass channel (p). The parameter that helps to ascertain and control the *QF* (β) is expressed as follows:

(20) $$QF=\frac{2-\beta }{\beta }$$

By implementing the iterative filter bank structure, the $k^{th}$ level of the FAWT is obtained. At every level, the decomposition in this technique provides dual channels and a single channel, which corresponds to high-pass channels and a low-pass channel, respectively.

The frequency response of the low-pass filter is expressed as follows:

(21) H(w)={(ul)12|w|<wr(ul)12θ(w−wrwu−wr)wr≤w≤wu(ul)12θ(π−(w−wr)wu−wr)−wu≤w≤−wr0|w|≥wu

The frequency response of the high-pass filter is expressed as follows:

(22) G(w)={(2ph)12θ(π−(w+w0w1−w0)w0≤w<w1(2ph)12w1≤w≤w2(2ph)12θ((w−w2w3−w2)w2≤w≤w30w∈[0,w0)∪(w3,2π)

where

(23) $$w_{r}=\frac{\left(1-\beta \right)\pi }{u}+\frac{e}{u}$$

(24) $$w_{u}=\frac{\pi }{l}$$

(25) $$w_{0}=\frac{(1-\beta )\pi +e}{p}$$

(26) $$w_{1}=\frac{u\pi }{lp}$$

(27) $$w_{2}=\frac{\pi -e}{p}$$

(28) $$w_{3}=\frac{\pi +e}{p}$$

(29) $$e\leq \frac{u-l+\beta t}{u+l}\pi$$

θ(w) is expressed as follows:

(30) θ(w)=[1+cos(w)[2−cos(w)]]122 for w∈[0,π]

The following conditions should be fulfilled so that a proper reconstruction is allowed:

(31) $${\left|\theta \left(\pi -w\right)\right|}^{2}+{\left|\theta (w)\right|}^{2}=1$$

(32) $$\left(1-\frac{u}{l}\right)\leq \beta \leq \frac{p}{h}$$

Without using the chirp parameters, the parameter of the FAWT is deduced. The value of β is set to 0.5, and the QF is set to 2 in our experiment.

### 2.5. Chirplet Transform

For non-stationary signals, the time–frequency domain specifications are expressed well with the chirplet transform [48]. Information on the amplitude variation with time is expressed by the time domain signal. Information on the amplitude variation with frequency is expressed by the frequency domain signal. With respect to both frequency and time, information on the variation in energy or amplitude is expressed by the CT. The generalized specification of the STFT and CWT is the chirplet transform, and it has numerous applications in the fields of biosignal processing, image processing, etc. q(s) is a speech emotion signal with s=1,2,…,S, and the total number of samples present in the speech signal q(s) is represented by S. Then, the chirplet transform of q(s) is expressed as follows:

(33) $$Q_{\gamma ,\sigma }(m,s)=\sum_{s=1}^{S}\tilde{q}(s)e^{-\frac{j2\pi ms}{S}}\chi_{\tau ,\gamma ,\sigma }^{*}(s)$$

where $\tilde{q}(s)$ represents the analytic signal of q(s) and is evaluated using the Hilbert transform. The analytic signal of q(s) is expressed as $\tilde{q}(s)=q(s)+jH\left[q\left(s\right)\right]$, where the Hilbert transform of the speech emotion q(s) is represented by H[q(s)]. The complex conjugate of χ is represented by the factor $\chi^{*}$. In the chirplet transform, the window function is $\chi_{\tau ,\gamma ,\sigma }^{*}(s)$, and it is expressed as follows:

(34) $$\chi_{\tau_{0},\gamma ,\sigma }^{*}(s)=w_{\sigma }(s-\tau_{0})e^{-\frac{\gamma }{2}{\left(s-\tau_{0}\right)}^{2}}$$

where the Gaussian window function is represented by the factor $w_{\sigma }(s-\tau_{0})$ and is expressed as follows:

(35) $$w_{\sigma }(s)=\frac{1}{\sigma \sqrt{2\pi }}e^{-\frac{s^{2}}{2\sigma^{2}}}$$

By utilizing the chirplet transform of the speech emotion signal, the time–frequency matrix is obtained, and it is mathematically expressed as follows:

(36) $$Q_{\gamma ,\sigma }(m,\tau_{0})=Q_{\gamma ,\sigma }^{R}(m,\tau_{0})+jQ_{\gamma ,\sigma }^{I}(m,\tau_{0})$$

where the real part of the time–frequency matrix $Q_{\gamma ,\sigma }(m,\tau_{0})$ is expressed as $Q_{\gamma ,\sigma }^{R}(m,\tau_{0})$, and the imaginary part of the time–frequency matrix $Q_{\gamma ,\sigma }(m,\tau_{0})$ is expressed as $Q_{\gamma ,\sigma }^{I}(m,\tau_{0})$. For the time–frequency matrix, the magnitude is expressed as follows:

(37) $$\left|Q_{\gamma ,\sigma }(m,\tau_{0})\right|=\sqrt{{\left[Q_{\gamma ,\sigma }^{R}(m,\tau_{0}\right]}^{2}+{\left[Q_{\gamma ,\sigma }^{I}(m,\tau_{0}\right]}^{2}}$$

### 2.6. Superlet Transform

A collection of wavelets with a high bandwidth is used by the superlet transform [49]. The excellent temporal resolution of the wavelength is combined geometrically so that a high frequency resolution is attained. A time–frequency analysis of the signal is performed using the STFT, and, hence, both time and frequency representations of the signals are specified. A quick localization of both the time and frequency is not attained by the generated features of the STFT because of the Heisenberg uncertainty principle. The drawbacks of the STFT are mitigated by the CWT, and, thus, at higher frequencies, a good temporal resolution is achieved [50]. Thus, DWT is utilized where the specification of the frequencies is determined as a power of two. There are only a limited number of constraints in both the STFT and CWT. A good frequency resolution is provided by the STFT, and a good temporal resolution is provided by the CWT [51]. At higher frequencies, a good frequency resolution with poor temporal resolution is provided by STFT, whereas a good relative temporal resolution is maintained by CWT throughout the spectrum but it can easily degrade in frequency resolution and becomes redundant with increasing frequency [52]. To obtain a high time–frequency resolution, different wavelets are utilized by the superlet transform. A good temporal but low frequency resolution is provided by wavelets with a smaller number of cycles, and a high frequency resolution is obtained by wavelets with a greater number of cycles, but a degraded temporal resolution is obtained. To obtain a super-resolution, both high temporal resolutions and high frequency resolutions are combined by the superlet [53]. For various applications such as biosignal processing, image processing, wireless communication, and electrical signal processing, superlets have been widely used. The Morlet is utilized as a mother wavelet by the superlet transform, which expresses a multi-resolution spectro-temporal specification. One of the most complex wavelets is the Morlet wavelet, and it is expressed as follows:

(38) $$\phi (t)=\left(e^{-jw_{0}t}-e^{-\frac{w_{0}^{2}}{2}}\right)e^{-\frac{t^{2}}{2}}$$

where the central frequency of the mother wavelet is expressed as $w_{0}$. The Morlet wavelet is almost like the Gabor transform in terms of its process. The scaling parameter helps to scale the window function in the Morlet transform. In the Gabor transform, the window size is already prefixed. The modified Morlet implemented in the superlet is expressed as follows:

(39) $$\psi_{w_{0},n}(t)=\frac{1}{D_{n}\sqrt{2\pi }}e^{-\frac{t^{2}}{2D_{n}^{2}}}e^{j2\pi w_{0}t}$$

The displacement parameter is expressed as $D_{n}$, where the time variance of the wavelet is regulated as follows:

(40) $$D_{n}=\frac{n}{S.D\times w_{0}}$$

It is generally inversely proportional to the frequency. A broad frequency response is obtained if the value of $D_{n}$ is small. A narrow frequency response is obtained if the value of $D_{n}$ is large. $D_{n}$ is adjusted so that the wave covers the full cycles within the standard deviation S.D of the Gaussian envelope. Different wavelets are used by the superlet with $w_{0}$ so that a better time–frequency representation is obtained. The mathematical specification of the superlet is as follows:

(41) $$SL_{w_{0},p}=\left\{\psi_{w_{0},n}|n=n_{1},n_{2},\ldots ,n_{p}\right\}$$

The order of the superlet that manages the wavelet is expressed by p. For every wavelet, the number of cycles is represented by $n_{1},n_{2},\ldots ,n_{p}$ in the superlet. In the wavelets of the superlet transforms, the number of cycles is selected additively or multiplicatively. The number of cycles is utilized and selected by making use of the multiplicity concept as follows:

(42) $$n_{i}=n\times i$$

Here, i=1,2,3,…,p. As far as the individual wavelet response is concerned, the response of the superlet to the speech emotion signal q(t) is specified by the geometric mean, and it is expressed as follows:

(43) $$G\left[SL_{w_{0},p}\right]=p\sqrt{\prod_{i=1}^{p}G\left[\Psi_{w_{0},n_{i}}\right]}$$

Here, the response of the $i^{th}$ wavelet of q(t) is represented as $\Psi_{w_{0},n_{i}}$.

For the Morlet, it is expressed as follows:

(44) $$G\left[\Psi_{w_{0},n_{i}}\right]=\sqrt{2}.q*\Psi_{w_{0},n_{i}}$$

The speech emotion signal is represented by q, and the complex convolution is represented by ∗. Superlet transforms are almost like CWT, except for the fact that the superlet transforms utilize superlets instead of wavelets, which are utilized in the CWT. A CWT is explained as a superlet that includes superlets of order 1. Superlets with a higher order provide a good indication of the signal. For a wide frequency, adaptive superlets are preferred. If the adaptive superlet is used with an order higher than 1, then a good improvement in the time–frequency domain of the signal is obtained.

## 3. Feature Selection Approaches

An important step in the machine learning pipeline is automatic feature selection. It involves selecting the most important features while eliminating the least important ones. To enhance the performance of any machine learning model and to reduce the computational overhead, automatic feature selection techniques are utilized. Novel feature selection techniques utilize new ideas to extract these relevant features and can employ a plethora of techniques to do it successfully. Using three various approaches—two filter approaches and a wrapper approach—feature selection can be carried out [54]. Filter algorithms employ the inherent properties of the dataset to estimate the priority of features. Wrapper algorithms assess the performance of a particular classifier with respect to the chosen features. Hybrid methods use the techniques of both the wrapper and filter algorithms; therefore, the number of candidate features is reduced. The general wrapper algorithm is implemented by using an optimization algorithm with a classifier. Depending on the training results of the classifier, the feature combination elements are fine tuned. The training phase and the optimization algorithm are time consuming, so wrapper algorithms sometimes seem to be less suited for high-dimensional datasets. Hybrid techniques that involve the combination of both the filter and wrapper algorithms are also time consuming and can be computationally expensive. When comparing wrapper and hybrid techniques, filter algorithms have several advantages, such as easy implementation and high efficiency; thus, in this work, initially, Overlapping Information Feature Selection (OIFS) is used to choose the features depending on the overlapping of samples concept to improve the feature selection efficiency [55]. Geometrical separation is ensured for every pair of categories in at least one feature dimension. Using an approximate equation, the computation of the overlapping matrices of every feature is analyzed. Global features are projected as the features that have the lowest overlapping rate. Then, the pair of categories that cannot be split by the global features is identified, and the pair with the lowest overlapping rate is considered. Following OIFS, the HHO algorithm and CSA algorithm are also utilized for the efficient selection of features in this work.

### 3.1. OIFS Method

For every class, the boundaries must be identified, and this serves as the main objective of a classification problem. When there is a geometrical distribution among the various boundaries of the classes, the problem becomes a straightforward one. Here, the overlapping rate of the sample is considered an important factor for the feature selection method [55]. If one feature is used to geometrically separate two classes, then a low overlap between these two classes is implied, thereby facilitating the easy discrimination of the features. For each pair of classes, if an appropriate feature satisfies the above condition, then the complexity is reduced. The approximation of the overlapping rate between two classes is expressed as follows:

(45) $$F\left(h_{1},h_{2}\right)=\frac{\sigma_{h_{1}}^{2}+\sigma_{h_{2}}^{2}}{{\left(\mu_{h_{1}}-\mu_{h_{2}}\right)}^{2}}$$

where $\mu_{h_{1}}$ and $\mu_{h2}$ represent the sample means, $\sigma_{h_{1}}$ and $\sigma_{h_{2}}$ represent the standard deviation of class $h_{1}$ and class $h_{2}$, respectively. For the N class classification issue, the overlapping matrix of feature g is expressed as follows:

(46) $$F_{g}=\left[\begin{array}{cccc} f_{g}(1,1) & f_{g}(1,2) & \ldots & f_{g}(1,N)\\ f_{g}(2,1) & f_{g}(2,2) & \ldots & f_{g}(2,1)\\ : & : & : & :\\ f_{g}(N,1) & f_{g}(N,2) & \ldots & f_{g}(N,N) \end{array}\right]$$

The overlapping matrix is considered symmetric in nature, and, using Equation (45), all the values of every element can be derived. It is not necessary to compute the diagonal elements, as they specify the overlapping rate of every class by itself. A feature set comprises $N_{f}$. Depending on these matrices, the features are selected. To distinguish every pair of classes, at least one feature is required. Overlapping matrices generally have a (N×N) structure. Training the overlapping matrices is the best method for choosing a feature for every pair of classes. The corresponding feature of the overlapping matrix is then selected. Ultimately, in the final feature combination, N(N−1)/2 features are chosen. Some redundant features may exist in the chosen combination. There are two important phases of OIFS, and they aim to mitigate the redundancy of feature combination. The first phase consists of choosing the global features so that multiple pairs of classes can be clearly discriminated against and computational efficiency is easily improved. This process can also aid in easy classification. Due to the inherent properties, some classes may show blurred boundaries, so local features must be used. The next phase in OIFS concentrates on choosing the local features so that the classification of two ambiguous classes can be very well designed and facilitated. To choose the global features, the procedure is as follows: The average overlapping rate is computed for every overlapping matrix, and, for the $g^{th}$ feature, it is represented as follows:

(47) $${\bar{F}}_{g}=\frac{\sum_{h_{1}=1}^{N}\sum_{h_{2}=1,h_{2}\neq h_{1}}^{N}f_{g}(h_{1},h_{2})}{N(N-1)}$$

The average value of the overlapping matrix is computed using Equation (47). Depending on the average overlapping rates, the features are sorted in ascending order. The global features consist of only the top features selected from this procedure. The number of candidates chosen as global features is identified as $N_{c}$. The input is converted to an integer using the function int(). $N_{z}(g)$ specifies the $g^{th}$ element of vector $N_{z}$. Using the $g^{th}$ feature, the pair of classes can be easily discriminated. For global features, the overlapping limit is indicated by $\varepsilon_{1}$. Here, the parameters q and $\varepsilon_{1}$ are considered tuning parameters, and their values are assigned as follows: q=10 and $\varepsilon_{1}=0.5$. The values of q and $\varepsilon_{1}$ are increased if the number of features is reduced; thus, the global features are increased, and the local features are decreased. The pair of classes that is not identified by the global features is traced out. Local features are assigned to these pair of classes, and the minimum overlapping rate is computed. In between the two related classes, the determination of a feature with the ability to assign itself to a high overlapping rate or low overlapping rate is ascertained. In the feature set, the minimum overlapping rate between the two classes is recorded so that the local features can be selected. The overlapping rate limit is ascertained and checked to ensure that it always satisfies the criterion. Several iterations are required to choose only the efficient features, thereby eliminating the redundant ones.

### 3.2. Harris Hawks Optimization

In nature, the associated and relative behavior of Harris Hawks influences the HHO algorithm [56]. Due to its versatility, it has been proven to be a promising search technique, and it is used to address a plethora of optimization issues. The HHO algorithm has two exploration stages and four exploitation stages. To improve the quality of the results, different intelligent schemes are employed, which utilize a greedy scheme.

#### 3.2.1. Stages of Initialization

In this stage, both the search spaces and fitness are presented. The basic chaotic opposition-dependent initialization technique is used, and all the parameters are assigned values.

#### 3.2.2. Stages of Exploration

In the HHO algorithm, Harris Hawks is tested as a candidate solution. Using two strategies, the fitness is computed depending on the planned prey, and this stage is expressed as follows:

(48) $$a_{t+1}=\{\begin{array}{c} a_{rand}(t)-r_{1}\left|a_{rand}(t)-2r2a(t)\right|\begin{array}{cc} & p\geq 0.5 \end{array}\\ a_{rabbit}(t)-a_{m}(t)-r_{3}\left(LB+r_{4}\left(UB-LB\right)\right)\begin{array}{cc} & p<0.5 \end{array} \end{array}$$

where the new position in the second iteration is expressed as $a_{t+1}$, and the current position is expressed as a(t). The random position of the hawk is indicated as $a_{rand}(t)$, and the optimal position of the intended rabbit is expressed as $a_{rabbit}(t)$. $r_{1},r_{2},r_{3},r_{4}$ and p specify the random numbers within [0, 1], and this is updated in every iteration. The lower bound is represented by LB, and the upper bound is represented by UB. The average position of the N solution is represented by $a_{m}(t)$, and it is expressed as follows:

(49) $$a_{m}(t)=\frac{1}{N}\sum_{i=1}^{N}a_{i}(t)$$

where the total number of hawks is denoted as N, and the position in iteration t of every hawk is represented as $a_{i}(t)$.

#### 3.2.3. Exploration Stage to Exploitation Stage

In this stage, when the escaping action occurs, the energy of the prey is mitigated; therefore, the exploration stage leads to the exploitation stage, and this is expressed as follows:

(50) $$E=2E_{0}\left(1-\frac{1}{T}\right)$$

where the escape energy of the prey is indicated by E, the starting stage of the energy is represented by $E_{0}$, and the maximum number of iterations is represented by T. The position of the prey is observed by the hawk in the case of |E|≥1, and this indicates the exploration phase of the HHO. In the case of |E|<1, the hawk is in the exploitation phase.

#### 3.2.4. Stages of Exploitation

This stage comprises four important steps: soft surrounding, hard surrounding, hard surrounding, hard surrounding, hard surrounding, hard surrounding with rapid dives, and soft surrounding with rapid dives. A summary of the steps is expressed as follows: A soft surrounding strategy occurs when r and |E|<0.5. The position of the hawk is updated as follows:

(51) $$a\left(t+1\right)=\Delta a(t)-E\left|Ja_{rabbit}(t)-a(t)\right|$$

(52) $$\Delta a(t)=a_{rabbit}(t)-a(t)$$

where the difference between the position vector of the rabbit and the present position in iteration t is expressed as Δa(t). The random jumping power of the rabbit is expressed by J=2(1−r5), where the random variable is expressed as r5. The hard surrounding strategy is considered if r≥0.5 and |E|<0.5. In this case, the position of the hawk is updated as follows:

(53) $$a(t+1)=a_{rabbit}(t)-E\left|\Delta a(t)\right|$$

A soft surrounding strategy with rapid dives occurs when |E|≤0.5 and r<0.5. In this stage, the prey successfully escapes, and the hawks must make a collection decision; this is expressed as follows:

(54) $$B(t)=a_{rabbit}(t)-E\left|Ja_{rabbit}\left|t\right|-a\left(t\right)\right|$$

To model this strategy, the best Levy flight motion-based patterns are utilized by HHO, and they are defined as follows:

(55) C=B+V×LF(D)

where the dimension of the solution is represented by D, a random number vector of size 1×D is represented by V, and the Levy flight motion is denoted by *LF*. *LF* is expressed as follows:

(56) $$LF(a)=0.01\times \frac{\mu \times \alpha }{{\left|w\right|}^{\frac{1}{\beta }}},$$

(57) α=(τ(1−β)×sin(πβ2)τ(1−β2)×β×2(1−β2))1β

where w is a random value in the range of [0, 1].

The constant is defined by β and is assigned a value of 2 in our work. The hawk’s position is updated as follows:

(58) $$a(t+1)=\{\begin{array}{c} B\begin{array}{cc} & if\begin{array}{cc} & F(B)<F\left(a\left(t\right)\right) \end{array} \end{array}\\ C\begin{array}{cc} & if\begin{array}{cc} & F(C)<F\left(a(t)\right) \end{array} \end{array} \end{array}$$

where B and C are utilized by using (54) and (55), and both equations refer to the locations of the next new iteration. A hard surrounding strategy with rapid dives is implemented if r<0.5 and |E|<0.5, and this is expressed as follows:

(59) $$a(t+1)=\{\begin{array}{c} B^{\prime }\begin{array}{cc} & if\begin{array}{cc} & F(B^{\prime })<F\left(a(t)\right) \end{array} \end{array}\\ C^{\prime }\begin{array}{cc} & if\begin{array}{cc} & F\left(C^{\prime }\right)<F{\left(a(t)\right)}^{\prime } \end{array} \end{array} \end{array}$$

where

(60) $$B^{\prime }(t)=a_{rabbit}(t)-E\left|Ja_{rabbit}(t)-a(t)\right|$$

(61) $$C^{\prime }=B^{\prime }+V\times LF(D)$$

#### 3.2.5. Initialization Based on Chaotic Opposition

The candidate solutions are randomly initialized, and the search process is started with the traditional optimization technique. The solution space of the population is initialized by the implementation of the chaotic map strategy. The convergence speed must be increased, so the chaotic opposition-dependent learning population technique replaces the random initialization solution. An initial state is generated by the chaotic opposition to improve the solution diversity, and this is achieved by utilizing a chaotic map to introduce randomness. Thus, the algorithm is prevented from prematurely converging to a local optimum, and the global search ability is greatly improved.

#### 3.2.6. Simulated Annealing

Simulated annealing is a very famous local search technique and is analyzed as a single heuristic dependent on solid annealing [57]. The issue of stagnation in the local economy can be surmounted by the application of this approach. With a specific probability, even a worse solution can be accepted by SA. The Boltzmann probability $e^{-\frac{\theta }{T}}$ controls the worst solution/worst neighbor, where the difference between the best solution and generated neighbor fitness is expressed by θ. During the search process, the temperature is indicated by T, and this is periodically decreased. The starting temperature is set to 2×|N|, where the number of attributes for every data point is expressed by |N|, and the cooling time is computed appropriately.

#### 3.2.7. Operators of Mutation and Crossover

Every Harris Hawk is placed in a random location in the exploration of HHO so that the location of the rabbit can be determined. The exploration stage of the feature space is improved, and the actual location updated point is detected so that the crossover and mutation can be well prevented as described below. The mutation rate is expressed as follows:

(62) $$b(t)=0.9+\frac{-0.9\times a(t-1)}{T-1}$$

where the maximum number is denoted by T, and the current iteration is expressed as t. As the total number of iterations increases, there is a decrease in mutation b, and this happens in a linear manner from 0.9 to 0. During the iteration procedure, the crossover is added between the current solution a(t) and the resultant value of the mutation, and this operation is expressed as follows:

(63) $$a{\left(t+1\right)}_{i}=\{\begin{array}{c} a{\left(t\right)}_{i}\begin{array}{cc} & p<0.5 \end{array}\\ a_{i}^{Mut}\begin{array}{cc} & p\geq 0.5 \end{array} \end{array}$$

where the value resulting from the mutation is expressed as $a^{Mut}$, a random number in the range [0, 1] is denoted as p, and the freshly generated solution is denoted by a(t+1). The $i^{th}$ size in A(t+1) is denoted as $a{\left(t+1\right)}_{i}$.

#### 3.2.8. Tournament Choosing

One useful selection strategy is tournament choosing, and it accesses a specific tournament among randomly chosen individuals from a specific object. There are four main phases in this strategy. The population size and tournament are utilized as input values; then, a random number r is generated in the range of [0, 1]. This random number is compared with that of the selection probability to adjust the selection pressure. If the fitness value is high, then the best solution is obtained if the tournament size is great; otherwise, the weak solution is selected. Ultimately, diversity is preserved if the tournament choosing strategy is utilized.

#### 3.2.9. KNN Classifier

**Figure 2:**
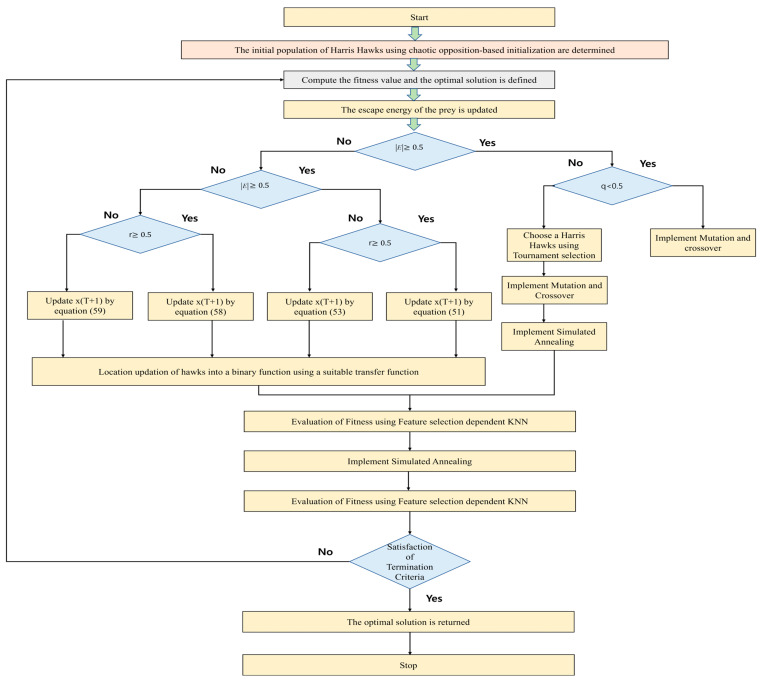
Feature selection method utilizing the optimization algorithm and KNN.

A versatile classification technique is KNN [58]. The main concept of this technique is that a particular sample refers to a specific group and the characteristics quite common to the samples present in the group are exhibited. If a high majority of K samples belong to that category, then classification occurs. This technique is used in signal processing, image processing, text classification, financial risk level assessment, etc., as it is quite straightforward, easy to understand, and simple to implement. The distance chosen in this study is the Euclidean distance, and the mathematical equation is as follows:

(64) $$dis\tan ce\left(G,H\right)=\sqrt{\sum_{K=1}^{N}{\left(G^{K}-H^{K}\right)}^{2}}$$

where the dimension of the sample is represented by N. The sample in the training set is indicated by G, and the sample in the test set is indicated by H. The feature selection method utilizing the optimization algorithm and KNN is shown in a flowchart in Figure 2.

#### 3.2.10. Parameter Settings of HHO

Experiments were carried out on a Window 10 computer with an i5 core processor and 8 GB memory. When using the KNN classifier, the Euclidean distance metrics’ K value was assigned 8 in our experiment. The results of various optimization algorithms were run over 50 times. The size of the population was set to 25, and the maximum number of iterations was set to 100. The value of r was set to 4, and the number of hawks was set to 10. The α value was fixed at 0.001, and the β value was fixed at 0.95. For the SA algorithm, the particle number was set to 20. The final temperature was set to 0.2, and the cooling plan was set to 0.5.

### 3.3. Standard Chameleon Swarm Algorithm (CSA)

The foraging behavior of the chameleons is characterized by the mathematical model [59] described below.

#### 3.3.1. Population Initialization

With a collection of ‘n’ potential solutions representing the number of chameleons, the position of chameleons z in the search space is randomly initialized, where a possible solution is specified by every chameleon. At a specific iteration t, the position of the $j^{th}$ chameleon in the search space is expressed as follows:

(65) $$z_{t}^{j}=\left[z_{t,1}^{j},z_{t,2}^{j},\ldots ,z_{t,d}^{j}\right]$$

where j=1,2,…,n, the current iteration is represented by t, and the dimension is represented by d. The position of chameleon j in a particular dimension d is represented by $z_{t,d}^{j}$. In the search space, the initial population of the Chameleon Swarm Algorithm is randomly generated depending on the total number of chameleons and the given problem dimension as expressed below:

(66) $$z^{j}=l_{k}+r\times \left(u_{k}-l_{k}\right)$$

where the initial vector of chameleon i is represented by $z^{i}$. In dimension k, the lower and upper limits of the search space are specified by $u_{k}$ and $l_{k}$, respectively. The random value, which is uniformly created, is represented by r and is in the range of [0, 1]. Predetermined fitness criteria are used to determine the new position solution for every chameleon. For every chameleon, the present position is updated if a solution with a better quality than the current position solution is identified. When the CSA algorithm is simulated, if the current solution quality is high and better than that of the new position, then the chameleon stays in its original position.

#### 3.3.2. Position Update

When searching for the prey, the positions of the chameleons are updated with the help of the position update strategy. The foraging conduct of the chameleon is represented as follows:

(67) $$z_{t+1}^{j,k}=\{\begin{array}{c} z_{t}^{j,k}+a_{1}\left(A_{t}^{j,k}-B_{t}^{k}\right)+a_{2}\left(B_{t}^{k}-z_{t}^{j,k}\right)r_{1}\begin{array}{cc} & r_{i}\geq Aa \end{array}\\ z_{t}^{j,k}+\mu \left(\left(u^{k}-l^{k}\right)r_{3}+l_{b}^{k}\right).\mathrm{sgn}\left(rand-0.5\right)\begin{array}{cc} & r_{i}\leq Aa \end{array} \end{array}$$

where the normal position of chameleon j at distance k and iteration t+1 is represented by $z_{t+1}^{j,k}$. The present position of chameleon j in dimension k and iteration t is represented by $z_{t}^{j,k}$. $A_{t}^{j,k}$ traces the highest and best position achieved by chameleon j in dimension k. $B_{t}^{k}$ represents the global best position achieved by the chameleon in dimension k. $a_{1}=0.5$ and $a_{2}=0.75$ are considered two positive values that help in assessing the exploration behavior. In the interval [0, 1], the random values are denoted *r*_1_, *r*_2_, and *r*_3_. The random value present in index i is denoted by $r_{i}$ and is in the range of [0, 1]. Aa denotes the discerning prey of the chameleon, and sgn(rand−0.5) is either −0.5 or +0.5 and can highly influence the exploitation/exploration phase. The function of the iterations is denoted as μ and is expressed as follows:

(68) λ=c1e(−c2×(tT))C3

where t represents the current number of iterations, and T indicates the maximum number of iterations. $c_{1},c_{2},c_{3}$ are constant values, with values of 1, 1.5, and 2, respectively, and they help to mitigate the exploitation behavior. Parameter $B_{t}^{k}$ indicates that the present candidate solution is highly similar to the optimal solution. The probability of a chameleon managing the prey in the environment is represented by parameter Aa, and Aa≥0.1. This condition is fixed so that the present position can be altered based on the prey observation in the search space. When searching for the prey, the locations in the search space are altered by the chameleons in a random manner. To identify prey, the search space is randomly explored in various areas and directions so that a high potential is achieved; thus, the optimal goals can be easily represented.

#### 3.3.3. Update Model Based on the Eye Rotation of the Chameleon

The chameleon possesses an innate ability to perceive the position of prey, as they are able to rotate their eyes to detect prey within a 360-degree range; this gives them special access and power. A chameleon can easily spin and move towards the prey’s position, and the novel position is represented as follows:

(69) $$z_{t+1}^{j}=r\times \left(z_{t}^{j}-{\bar{z}}_{t}^{j}\right)+{\bar{z}}_{t}^{j}$$

where the updated position of the chameleon is represented by $z_{t+1}^{j}$, and the rotation matrix of the chameleon is represented by r. The current position of the chameleon is represented by $z_{t}^{j}$, and the center of the chameleon’s present position is represented as ${\bar{z}}_{t}^{j}$, which is determined as follows:

(70) $$r=R\left(\theta ,{\vec{o}}_{1},{\vec{o}}_{2}\right)$$

where the orthonormal vectors are represented as ${\vec{o}}_{1}$ and ${\vec{o}}_{2}$, and they have a size of d×1. The rotation matrices are indicated by R, and the cycle of rotation of the eyes is denoted by θ, which can be expressed as follows:

(71) $$\theta =ra.\mathrm{sgn}\left(rand-0.5\right)\times 180^{{}^{\circ}}$$

where ra denotes the rotation angle from 0 to 180 degrees, which is in an interval of [0, 1]. sgn(rand−0.5) is either −1 or +1, and it denotes the rotation direction.

#### 3.3.4. Velocity Update Model

The chameleon terminates the stalking process once the prey is assaulted and it is not far from the chameleon’s position. This chameleon is considered the best among all the chameleons. The position of this chameleon is updated, as it can extend its tongue to twice its length. This helps the chameleons catch the prey efficiently, as it allows them to exploit the search area quickly. In this algorithm, the chameleon’s tongue moves towards the prey rapidly, and the velocity of this can be modeled as follows:

(72) $$v_{t+1}^{j,k}=wv_{t}^{j,k}+c_{1}\left(B_{t}^{k}-z_{t}^{j,k}\right)r_{1}+c_{2}\left(A_{t}^{j,k}-z_{t}^{j,k}\right)r_{2}$$

In dimension k, iteration t, and t+1, the following and current velocities of chameleon j is represented by $v_{t+1}^{j,k}$ and $v_{t}^{j,k}$. The present position of chameleon i is indicated by $z_{t}^{j,k}$. The two random values are denoted by *r*_1_ and *r*_2_ in an interval of [0, 1]. To manage the effects of $A_{t}^{j,k}$ and $B_{t}^{k}$ on the chameleon’s tongue, the position values *c*_1_ and *c*_2_ are considered important. The inertia weight is indicated by w and is determined as follows:

(73) $$w=\left(1-t/T\right)\left(\rho \sqrt{\left(t/T\right)}\right)$$

where, to mitigate the exploitation features, the positive values are utilized as ρ.

When moving towards the prey, the position of the chameleon’s tongue is computed as follows:

(74) $$z_{t+1}^{j,k}=z_{t}^{j,k}+\left({\left(v_{t}^{j,k}\right)}^{2}-{\left(v_{t-1}^{j,k}\right)}^{2}\right)/\left(2a\right)$$

where $v_{t-1}^{j,k}$ denotes the former velocity of chameleon j and dimension k. The rate of acceleration is denoted by a, and it reaches an approximate value of 2600 m/s, as shown below:

(75) $$a=2600\times \left(1-e^{-\log(t)}\right)$$

By randomly generating the chameleon position, the CSA performs optimization and feature selection. Within every iteration, the positions of all the chameleons are consecutively updated. The chameleons are returned to the boundary if they exist in the search space. With the help of fitness functions, solutions are assigned so that the fittest chameleon is identified. In every loop, the steps are reiterated until the iteration condition is met. The chameleon incessantly explores and exploits the search space so that they can move towards the prey and catch it with their tongue. The mathematical model provided above helps to address the optimization issue, even with very large search spaces. The simplified implementation of the CSA algorithm is explained in Algorithm 1.
**Algorithm 1:** Implementation of CSA algorithmThe Key parameters of CSA are assigned.The position for all chameleons is analyzed using u and l, then initialized.The velocity of falling chameleon’s tongue is assigned.Evaluate the starting position of all chameleonsWhile (*t* < *T*) do    Update the position of chameleons based on Equation (67)    Update the position of chameleons depending on their eyes turn merit using Equation (69)    Update the tongue velocity of the chameleon using Equation (72)    Update the tongue position of the chameleon using Equation (74)    Integrate the position of chameleons using u and l of the problem variables.    Update the position of every chameleon    *t* = *t* + 1end whileReturn the global best position of the chameleons.

#### 3.3.5. Parameter Settings of CSA

We conducted experiments on a Window 10 computer with an i5 core processor and 8 GB memory. The fitness was evaluated using fitness-dependent KNN with Euclidean distance metrics, and K was assigned a value of 8. The results of various optimization algorithms were run over 50 times. The size of the population was set to 25, and the maximum number of iterations was set to 100. The α value was fixed at 0.99, and the β value was fixed at 0.05.

## 4. Classification Using Machine Learning Algorithms

In this study, ten machine learning algorithms were employed, as described below.

### 4.1. Random Forest (RF)

Multiple decision trees are constructed randomly by means of sampling with replacement. The bagging technique is adopted by random forest, where many weak learners are integrated to form a strong learner. Weak learners are generated in an independent manner and based on the majority votes of the prediction of the weak learners, the final prediction is obtained.

### 4.2. Support Vector Machine (SVM)

SVM is a famous machine learning algorithm utilized for classification and regression problems. The margin is maximized around the separation bounds of two classes in a classification problem using a plane, line, or hyperplane to split the data.

### 4.3. Extreme Gradient Boosting (XGB)

XGB is quite an efficient, portable, and flexible parallel tree boosting system. The generalization issue is overcome by XGB, and the modal complexity is greatly reduced by the addition of a regularization parameter to the objective function.

### 4.4. Naïve Bayesian Classifier (NBC)

NBC depends on Bayes’ theorem learning technique. The conditional probability of every point is computed based on the conditions of every class. The class of the test data is like a class with a high conditional probability. Every feature has a strong independence, and there is no correlation between them in the NBC.

### 4.5. Gradient Boosting

Gradient boosting is a famous gradient descent-dependent boosting technique. Here, gradient descent is used so that the errors of the individuals are minimized. In an iterative manner, base learners are constructed by means of reweighting the misclassified instances. In every training observation, the negative partial derivatives of the loss function are operated by the gradient boosting to determine the weights. To obtain a high-performance model, weak learners are combined sequentially.

### 4.6. K Nearest Neighbor (KNN)

The KNN algorithm is a famous non-parametric technique. The K-nearest training points are used in the test so that the class value of the test points can be easily predicted. KNN is suitable for a high amount of training data, and it is not influenced by the noise of the training dataset.

### 4.7. Adaptive Boosting (AB)

Adaptive boosting (AB) is a famous boosting classifier, and, here, multiple base decision tree classifiers are combined in order to create a robust classifier with a good classification accuracy. Using weighted data, a sequence of weak classifiers is trained iteratively, and an ensemble is constructed by focusing on prior misclassified cases. Depending on the weighted summation of all weak classifiers, the final boosted classifiers are determined.

### 4.8. Decision Trees (DTs)

DT comprises a non-parametric tree-like structure with a root, leaf, and internal nodes. The training dataset is divided into many non-overlapping data subsets through the Gini Index. Depending on the attribute value, a partition is made by the root node, features are indicated by the internal nodes, and the outcome is specified by the leaf node.

### 4.9. Extreme Learning Machine (ELM)

For the training set $T=\left\{\left(a_{i},b_{i}\right):a_{i}\in {ℜ}^{n}\right\},$$b_{i}\in \left\{1,-1\right\},i=1,2,\ldots ,N$, and the number of hidden nodes is represented by L. The following optimization is solved using the standard ELM:

(76) $$\underset{\left(\beta ,\xi \right)}{\min}\frac{1}{2}{\left\|\beta \right\|}^{2}+\frac{c}{2}\sum_{i=1}^{N}\xi_{i}^{2}$$

This is subject to

(77) $$h(a_{i})=\left[J\left(w_{1},s_{1},a_{i}\right),J\left(w_{2},s_{2},a_{i}\right),\ldots ,J\left(w_{L},s_{L},a_{i}\right)\right]$$

which denotes the output vector of the hidden layer for input $a_{i}$, where all weights $w_{i}$ and biases $b_{i}$ are randomly selected. A user-defined parameter is expressed as c≥0 so that a tradeoff is achieved between the regularization and the empirical error. The optimization problem of the ELM is expressed as follows:

(78) $$\underset{\left(\beta \right)}{\min}\frac{1}{2}{\left\|\beta \right\|}^{2}+\frac{c}{2}{\sum_{i=1}^{N}\left(h\left(a_{i}\right)\beta -b_{i}\right)}^{2}$$

For the output weight vector β, the optimal value is expressed as follows:

(79) $$\hat{\beta }=H^{†}T=\{\begin{array}{c} H^{T}{\left(\frac{I}{C}+HH^{T}\right)}^{-1}B\begin{array}{cc} & if\begin{array}{cc} & N<L \end{array} \end{array}\\ {\left(\frac{I}{C}+HH^{T}\right)}^{-1}H^{T}Y\begin{array}{cc} & if\begin{array}{cc} & N\geq L \end{array} \end{array} \end{array}$$

where the Moore–Penrose generalized inverse is expressed as $H^{†}$, and the least norm least squares solution is obtained [60]. For the binary classification issue, the output function of the ELM considering the optimal weight β is analyzed as follows:

(80) $$f(a)=sign\left(\sum_{i=1}^{L}\beta_{i}J\left(w_{i},s_{i},a\right)\right)=sign\left(h\left(a\right)\beta \right)$$

In the ELM optimization issue, the least squares loss function is used so that it is made sensitive to noise in the data. When working with imbalanced datasets, poor performance is obtained when using the standard ELM. To manage class-imbalanced datasets efficiently, many variants of the ELM model have been developed. Various schemes are utilized by these variants so that the training points are weighed based on their corresponding class representation. In the past decade, deep learning has also been coupled with the ELM, as the iterative tuning of weights is not required. Some famous examples include the residual ELM, stacked ELM autoencoder, and local receptive field ELM.

### 4.10. Twin ELM

A twin ELM is used to obtain a better classification accuracy. A pair of quadratic programming problems are solved by the TELM, and two non-parallel hyperplanes are obtained in a random feature space [61].

(81) $$f_{1}(a):=h(a)\beta_{1}=0\;\mathrm{and}\;f_{2}(a):=h(a)\beta_{2}=0$$

The following pairs of QPPs are solved using the TELM model:

(82) $$\underset{\beta ,\xi }{\min}\frac{1}{2}{\left\|Y\beta_{1}\right\|}^{2}+c_{1}e_{2}^{T}\xi$$

This is subject to

(83) $$-Z\beta_{1}+\xi \geq e_{2},\xi \geq 0$$

(84) $$\mathrm{and}\underset{\beta ,\eta }{\min}\frac{1}{2}{\left\|Z\beta_{2}\right\|}^{2}+c_{2}e_{1}^{T}\eta$$

which is subject to

(85) $$Y\beta_{2}+\eta \geq e_{1},\eta \geq 0$$

The user-defined parameters are $c_{1}>0$ and $c_{2}>0$.

The corresponding Wolfe dual problems are obtained to derive efficient solutions to the primal problems, and they are expressed as follows:

(86) $$\underset{\alpha }{\max}e_{2}^{T}\alpha -\frac{1}{2}\alpha^{T}Z{\left(Y^{T}Y+\in I\right)}^{-1}Z^{T}\alpha$$

This is subject to $0\leq \alpha \leq c_{1}e$ and

(87) $$\underset{\gamma }{\max}e_{1}^{T}\gamma -\frac{1}{2}\gamma^{T}Y{\left(Z^{T}Z+\in I\right)}^{-1}Y^{T}\gamma$$

which is subject to $0\leq \gamma \leq c_{2}e$. The vectors of the Lagrange multiplier are denoted as α and γ.

## 5. Results and Discussion

The proposed work is tested on four robust datasets: the EMOVO [11], RAVDESS [12], SAVEE [13], and Berlin Emo-DB datasets [14]. EMOVO is an Italian speech database, and Berlin Emo-DB contains speeches made by German actors. The language of the SAVEE and RAVDESS datasets is English. There are seven emotions in the EMOVO, SAVEE, and Emo-DB datasets, while the RAVDESS speech dataset contains eight emotions. The EMOVO dataset consists of six actors (three males and three females) and about 588 observations, with a sampling rate of approximately 48 KHz. The RAVDESS dataset consists of 24 actors (12 males and 12 females) and about 1440 observations, with a sampling rate of approximately 48 KHz. The SAVEE dataset consists of four males and 480 observations, with a sampling rate of approximately 44.1 KHz. The EMO-DB dataset consists of 10 actors (5 males and 5 females) and 535 observations, with a sampling rate of approximately 16 KHz. A 10-fold cross validation method is used in our experiment. The proposed techniques are implemented and the results are obtained as described below. Table 1 shows a performance analysis of the classifiers with the transforms for the OIFS technique on the EMOVO dataset. A high classification accuracy of 78.65% is obtained for the FAWT with the twin ELM classification method. Table 2 shows a performance analysis of the classifiers with the transforms for the HHO feature selection technique on the EMOVO dataset. A high classification accuracy of 79.98% is obtained for the superlet transform with the twin ELM classification method. Table 3 shows a performance analysis of the classifiers with the transforms for the CSA feature selection technique on the EMOVO dataset. A high classification accuracy of 80.63% is obtained for the chirplet transform with the twin ELM classification method. Table 4 shows a performance analysis of the classifiers with the transforms for the OIFS technique on the RAVDESS dataset. A high classification accuracy of 84.19% is obtained for the FAWT with the twin ELM classification method. Table 5 shows a performance analysis of the classifiers with the transforms for the HHO feature selection technique on the RAVDESS dataset. A high classification accuracy of 85.76% is obtained for the FAWT with the twin ELM classification method. Table 6 shows a performance analysis of the classifiers with the transforms for the CSA feature selection technique on the RAVDESS dataset. A high classification accuracy of 85.54% is obtained for the chirplet transform with the twin ELM classification method. Table 7 shows a performance analysis of the classifiers with the transforms for the OIFS technique on the SAVEE dataset. A high classification accuracy of 83.94% is obtained for the chirplet transform with the twin ELM classification method. Table 8 shows a performance analysis of the classifiers with the transforms for the HHO feature selection technique on the SAVEE dataset. A high classification accuracy of 83.21% is obtained for the chirplet transform with the twin ELM classification method. Table 9 shows a performance analysis of the classifiers with the transforms for the CSA feature selection technique on the SAVEE dataset. A high classification accuracy of 83.11% is obtained for the KSTDIS transform with the twin ELM classification method. Table 10 shows a performance analysis of the classifiers with the transforms for the OIFS technique on the Berlin Emo-DB dataset. A high classification accuracy of 87.46% is obtained for the superlet transform with the twin ELM classification method. Table 11 shows a performance analysis of the classifiers with the transforms for the HHO feature selection technique on the Berlin Emo-DB dataset. A high classification accuracy of 87.57% is obtained for the KSTDIS transform with the twin ELM classification method. Table 12 shows a performance analysis of the classifiers with the transforms for the CSA feature selection technique on the Berlin Emo-DB dataset. A high classification accuracy of 89.77% is obtained for the KSTDIS transform with the twin ELM classification method.

**Figure 3:**
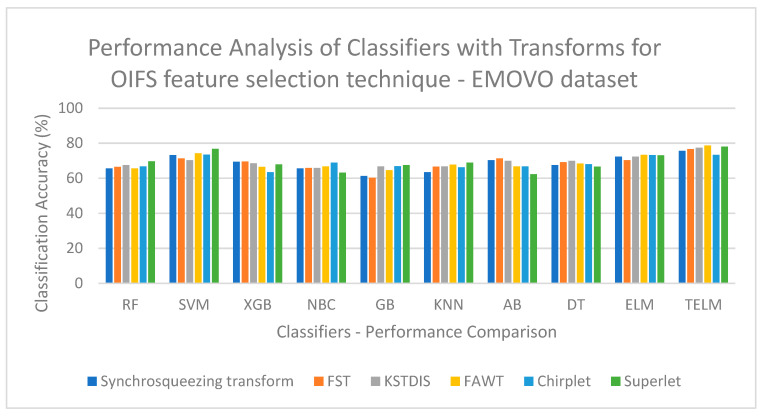
Performance analysis of classifiers with transforms for OIFS technique on EMOVO dataset.

**Figure 4:**
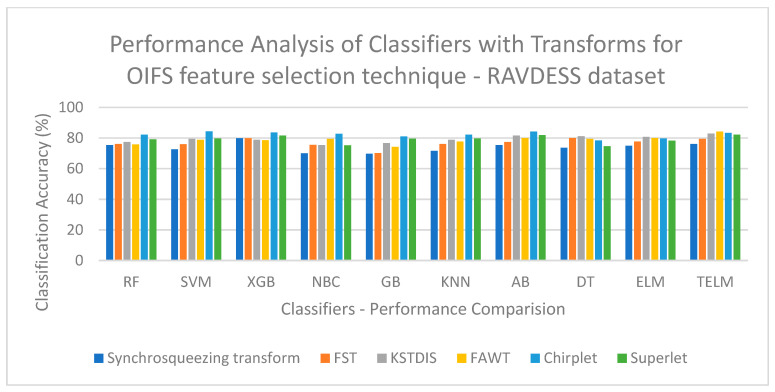
Performance analysis of classifiers with transforms for OIFS technique on RAVDESS dataset.

**Figure 5:**
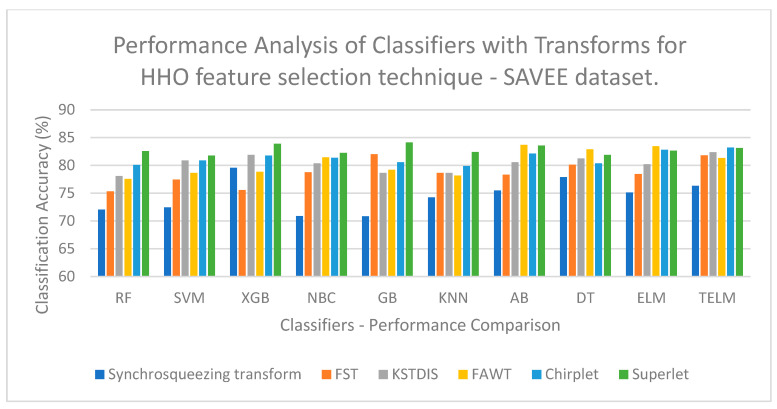
Performance analysis of classifiers with transforms for HHO feature selection technique on SAVEE dataset.

**Figure 6:**
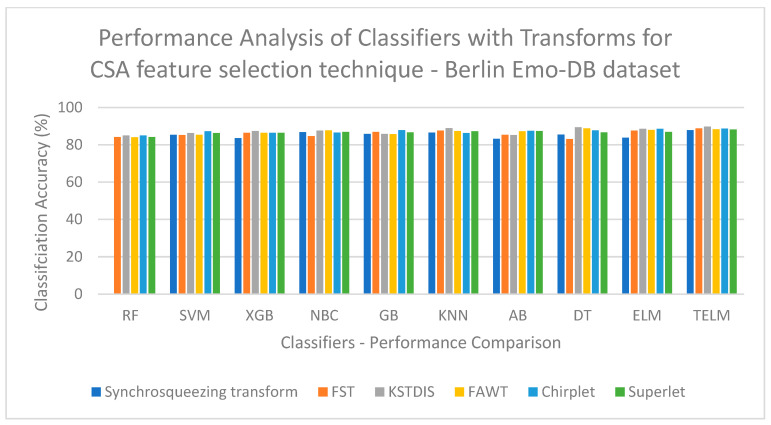
Performance analysis of classifiers with transforms for CSA feature selection technique on Berlin EMO-DB dataset.

Figure 3 displays a performance analysis of the classifiers with the transforms for the OIFS technique on the EMOVO dataset. Figure 3 shows that a low classification accuracy of 60.23% is obtained when the FST is implemented with the GB classifier. Figure 4 displays a performance analysis of the classifiers with the transforms for the OIFS technique on the RAVDESS dataset. Figure 4 shows that a low classification accuracy of 69.76% is obtained when the synchrosqueezing transform is implemented with the GB classifier. Figure 5 displays a performance analysis of the classifiers with the transforms for the HHO feature selection technique on the SAVEE dataset. Figure 5 shows that a low classification accuracy of 70.84% is obtained when the synchrosqueezing transform is implemented with the GB classifier. Figure 6 displays a performance analysis of the classifiers with the transforms for the CSA feature selection technique on the Berlin Emo-DB dataset. Figure 6 shows that a low classification accuracy of 83.12% is obtained when the FST is implemented with the DT classifier.

### Comparison with Previous Works

The results obtained here are compared with previously obtained results for all four databases, and the results are tabulated in Table 13, Table 14, Table 15 and Table 16.

An analysis of Table 13 shows that the proposed works produced very good results when compared with the previous results. The best classification accuracy result of 80.63% was obtained for the Chirplet + CSA + TELM combination on the EMOVO dataset. An analysis of Table 14 shows that the proposed works once again produced very good results when compared with the previous works. A high classification accuracy of 85.76% was obtained for the FAWT + HHO + TELM combination on the RAVDESS dataset. An analysis of Table 15 shows that a high classification accuracy of 83.94% was obtained for the Chirplet + OIFS + TELM combination on the SAVEE dataset, which is good when compared with previous works. An analysis of Table 16 shows that a high classification accuracy of 89.77% was obtained for the KSTDIS + CSA + TELM combination on the Berlin Emo-DB dataset, surpassing the results of previous works.

## 6. Conclusions and Future Works

SER is considered an important aspect of automatic speech recognition, where procedures such as feature extraction, feature selection, and classification using machine learning and deep learning seem to be the general norm. SER is an important area of research for the advancement of human–computer interactions and human–robot interactions. For automated emotion recognition, the most common data sources are physiological data, obtained from EEG and wearable sensors, and speech-based data. The fusion of single modal data or multimodal data can also be carried out. In this work, transform-dependent optimization techniques with machine learning classifiers are utilized for the classification of speech emotion, and the highest classification accuracy of 89.77% is obtained when the KSTDIS + CSA + TELM combination is applied to the Berlin Emo-DB dataset. The highest classification accuracy of 80.63% is obtained when the Chirplet + CSA + TELM combination is applied to the EMOVO dataset, the highest classification accuracy of 85.76% is obtained when the FAWT + HHO + TELM combination is applied to the RAVDESS dataset and the highest classification accuracy of 83.94% is obtained when the Chirplet + OIFS + TELM combination is applied to the SAVEE dataset. The proposed combinations can be applied for emotion detection in various domains. Future works include extending the methods to different types of speech-related pathologies. Moreover, the implementation of more efficient transforms coupled with optimization and machine learning/deep learning algorithms like Graph Neural Networks (GNN), Variational Autoencoders (VAE), Deep Belief Networks (DBN), Generative Adversarial Networks (GAN) and Transformer Networks is planned as future works so that a higher classification accuracy can be obtained. Future works also plan to implement this work in telemedicine applications and cloud-based domains so that remote telehealth services can be largely improved.

## Figures and Tables

**Table 1:** Performance analysis of classifiers’ accuracy with transforms for OIFS technique on EMOVO dataset.

	Feature Extraction through Transformation Techniques					
	Synchrosqueezing Transform	FST	KSTDIS	FAWT	Chirplet	Superlet
RF	65.56	66.45	67.45	65.54	66.78	69.65
SVM	73.23	71.23	70.23	74.21	73.45	76.78
XGB	69.34	69.45	68.56	66.41	63.45	67.89
NBC	65.56	65.78	65.78	66.78	68.89	63.23
GB	61.23	60.23	66.78	64.58	66.79	67.45
KNN	63.45	66.55	66.78	67.78	66.23	68.89
AB	70.23	71.23	69.91	66.67	66.69	62.34
DT	67.45	69.12	69.90	68.41	68.02	66.58
ELM	72.34	70.23	72.34	73.34	73.21	73.02
TELM	75.56	76.56	77.43	78.65	73.35	78.01

**Table 2:** Performance analysis of classifiers’ accuracy with transforms for HHO feature selection technique on EMOVO dataset.

	Feature Extraction through Transformation Techniques					
	Synchrosqueezing Transform	FST	KSTDIS	FAWT	Chirplet	Superlet
RF	66.23	65.56	68.33	66.09	62.09	68.09
SVM	75.56	72.78	72.45	75.87	70.89	77.68
XGB	70.87	70.98	69.67	68.77	61.78	68.32
NBC	67.96	68.44	69.87	69.75	62.55	68.45
GB	63.34	63.34	69.76	66.43	63.45	69.78
KNN	64.57	64.56	68.21	69.24	68.11	69.98
AB	72.89	74.89	66.34	69.68	62.27	65.23
DT	68.33	67.22	67.56	69.98	65.89	68.44
ELM	75.78	73.12	70.78	71.23	77.67	77.57
TELM	74.90	72.57	72.90	70.19	74.89	79.98

**Table 3:** Performance analysis of classifiers’ accuracy with transforms for CSA feature selection technique on EMOVO dataset.

	Feature Extraction through Transformation Techniques					
	Synchrosqueezing Transform	FST	KSTDIS	FAWT	Chirplet	Superlet
RF	69.23	69.90	69.09	69.89	66.66	68.09
SVM	78.45	75.98	75.89	79.98	74.78	77.98
XGB	73.67	78.77	73.88	72.78	66.98	68.78
NBC	69.87	69.65	74.76	73.67	67.54	68.67
GB	69.55	69.34	73.45	72.44	67.22	69.46
KNN	68.43	69.56	75.34	74.32	69.34	69.33
AB	77.12	79.43	69.23	73.13	67.56	65.21
DT	69.34	68.21	72.11	74.45	69.78	68.45
ELM	79.34	78.11	74.23	75.67	79.98	77.67
TELM	78.78	80.23	75.67	74.78	80.63	79.89

**Table 4:** Performance analysis of classifiers’ accuracy with transforms for OIFS technique on RAVDESS dataset.

	Feature Extraction through Transformation Techniques					
	Synchrosqueezing Transform	FST	KSTDIS	FAWT	Chirplet	Superlet
RF	75.34	76.09	77.33	75.78	82.09	79.09
SVM	72.67	75.89	79.44	78.76	84.34	79.77
XGB	79.88	79.88	78.78	78.55	83.55	81.63
NBC	69.98	75.43	75.34	79.43	82.78	75.24
GB	69.76	70.11	76.67	74.21	80.94	79.56
KNN	71.54	76.12	78.87	77.68	82.22	79.77
AB	75.34	77.38	81.63	79.97	84.12	81.87
DT	73.58	79.98	81.21	79.46	78.45	74.56
ELM	74.92	77.67	80.68	79.97	79.76	78.22
TELM	76.11	79.45	82.95	84.19	83.33	82.13

**Table 5:** Performance analysis of classifiers’ accuracy with transforms for HHO feature selection technique on RAVDESS dataset.

	Feature Extraction through Transformation Techniques					
	Synchrosqueezing Transform	FST	KSTDIS	FAWT	Chirplet	Superlet
RF	78.67	79.88	79.09	78.98	83.09	82.11
SVM	75.89	77.98	78.87	79.77	82.22	82.23
XGB	78.88	78.67	79.66	79.67	82.34	84.45
NBC	71.76	78.44	74.78	83.45	84.67	82.87
GB	72.44	73.32	79.98	79.67	81.87	84.64
KNN	73.34	77.13	79.22	82.89	80.54	83.22
AB	77.56	79.67	80.34	83.22	81.33	84.34
DT	78.75	77.87	80.56	82.23	79.23	80.78
ELM	76.11	78.69	82.76	83.45	80.45	80.96
TELM	75.23	78.87	84.11	85.76	84.67	84.11

**Table 6:** Performance analysis of classifiers’ accuracy with transforms for CSA feature selection technique on RAVDESS dataset.

	Feature Extraction through Transformation Techniques					
	Synchrosqueezing Transform	FST	KSTDIS	FAWT	Chirplet	Superlet
RF	79.03	80.98	82.11	81.22	82.09	81.11
SVM	78.44	79.89	84.23	82.34	81.99	84.26
XGB	79.56	79.76	83.78	83.56	83.87	85.08
NBC	74.78	80.48	84.73	85.07	82.56	84.93
GB	75.98	77.71	84.24	83.77	80.73	85.22
KNN	78.22	79.23	83.57	85.15	83.48	85.01
AB	79.12	82.48	81.65	84.41	85.05	81.68
DT	75.34	79.90	82.77	84.99	82.67	82.98
ELM	79.58	82.03	84.89	85.08	84.98	83.32
TELM	79.87	84.23	85.02	85.24	85.54	85.35

**Table 7:** Performance analysis of classifiers’ accuracy with transforms for OIFS technique on SAVEE dataset.

	Feature Extraction through Transformation Techniques					
	Synchrosqueezing Transform	FST	KSTDIS	FAWT	Chirplet	Superlet
RF	74.03	78.76	79.09	76.55	81.09	81.11
SVM	70.44	78.56	81.88	79.67	82.67	82.14
XGB	78.56	77.77	82.76	79.87	82.83	82.56
NBC	68.78	79.89	82.23	80.22	80.45	83.87
GB	72.97	80.93	79.45	77.12	81.67	83.23
KNN	72.34	79.21	79.67	79.34	80.88	80.56
AB	77.24	79.22	82.87	82.68	83.91	80.78
DT	76.67	81.45	80.22	81.96	79.35	78.44
ELM	77.87	79.67	81.11	82.31	80.78	79.31
TELM	78.23	82.87	83.25	83.74	83.94	82.03

**Table 8:** Performance analysis of classifiers’ accuracy with transforms for HHO feature selection technique on SAVEE dataset.

	Feature Extraction through Transformation Techniques					
	Synchrosqueezing Transform	FST	KSTDIS	FAWT	Chirplet	Superlet
RF	72.03	75.32	78.09	77.55	80.09	82.56
SVM	72.44	77.44	80.88	78.67	80.88	81.77
XGB	79.57	75.56	81.87	78.87	81.75	83.87
NBC	70.89	78.78	80.35	81.43	81.34	82.22
GB	70.84	81.98	78.67	79.22	80.57	84.12
KNN	74.23	78.65	78.66	78.15	79.86	82.40
AB	75.47	78.32	80.54	83.67	82.12	83.57
DT	77.89	80.11	81.23	82.87	80.34	81.87
ELM	75.12	78.45	80.21	83.44	82.78	82.65
TELM	76.32	81.78	82.34	81.31	83.21	83.12

**Table 9:** Performance analysis of classifiers’ accuracy with transforms for CSA feature selection technique on SAVEE dataset.

	Feature Extraction through Transformation Techniques					
	Synchrosqueezing Transform	FST	KSTDIS	FAWT	Chirplet	Superlet
RF	73.78	74.08	76.87	79.11	79.08	82.02
SVM	73.66	79.77	82.67	81.23	82.67	80.34
XGB	78.54	77.89	83.06	79.45	80.56	82.56
NBC	72.34	79.65	79.78	82.76	82.78	81.87
GB	72.67	80.34	76.98	82.87	81.76	82.65
KNN	75.87	75.56	79.23	80.43	82.43	80.32
AB	76.23	82.78	77.45	82.21	80.21	82.13
DT	78.12	82.62	80.67	81.23	79.65	80.67
ELM	74.34	81.13	78.87	80.56	81.78	81.66
TELM	75.67	79.46	83.11	82.76	82.87	82.99

**Table 10:** Performance analysis of classifiers’ accuracy with transforms for OIFS technique on Berlin Emo-DB dataset.

	Feature Extraction through Transformation Techniques					
	Synchrosqueezing Transform	FST	KSTDIS	FAWT	Chirplet	Superlet
RF	84.02	79.87	84.02	79.01	84.02	84.21
SVM	80.34	79.32	83.24	81.22	85.33	86.32
XGB	81.77	80.34	85.67	81.36	86.56	86.56
NBC	82.89	79.57	84.87	82.87	84.87	84.87
GB	80.78	81.86	83.21	79.43	85.54	85.32
KNN	79.22	82.43	84.23	83.21	82.22	86.44
AB	78.34	83.21	83.55	84.55	85.13	85.56
DT	80.67	84.02	85.67	83.78	86.54	85.87
ELM	82.97	85.34	83.89	85.98	83.76	86.21
TELM	83.12	84.47	86.21	86.22	86.89	87.46

**Table 11:** Performance analysis of classifiers’ accuracy with transforms for HHO feature selection technique on Berlin Emo-DB dataset.

	Feature Extraction through Transformation Techniques					
	Synchrosqueezing Transform	FST	KSTDIS	FAWT	Chirplet	Superlet
RF	83. 02	81.45	83.09	81.05	83.11	85.02
SVM	82.34	80.56	85.54	83.60	84.23	85.31
XGB	80.56	81.76	84.21	84.72	87.45	87.44
NBC	83.87	82.23	85.34	83.86	85.76	85.67
GB	82.54	82.23	82.66	82.22	86.54	86.89
KNN	79.23	83.46	86.78	84.11	84.21	85.87
AB	80.14	84.78	82.98	85.41	86.34	86.64
DT	82.56	82.98	87.22	83.56	85.78	84.13
ELM	83.77	86.21	86.12	86.78	85.65	85.67
TELM	84.89	85.22	87.57	85.97	87.19	86.87

**Table 12:** Performance analysis of classifiers’ accuracy with transforms for CSA feature selection technique on Berlin Emo-DB dataset.

	Feature Extraction through Transformation Techniques					
	Synchrosqueezing Transform	FST	KSTDIS	FAWT	Chirplet	Superlet
RF	85. 02	84.21	85.02	84.02	85.02	84.21
SVM	85.34	85.23	86.32	85.33	87.31	86.33
XGB	83.56	86.45	87.41	86.45	86.44	86.46
NBC	86.77	84.67	87.56	87.68	86.56	86.87
GB	85.87	86.89	85.78	85.76	87.87	86.64
KNN	86.53	87.65	88.98	87.41	86.33	87.21
AB	83.23	85.32	85.27	87.23	87.45	87.34
DT	85.45	83.12	89.34	88.78	87.78	86.70
ELM	83.78	87.56	88.56	87.98	88.54	86.87
TELM	87.91	88.78	89.77	88.31	88.67	88.21

**Table 13:** Performance comparison of classification accuracy—EMOVO dataset.

Authors	Concept Used	Number of Classes	Classification Accuracy (%)
Assuncao et al. [15]	Speaker awareness for SER techniques	7	68.50
Haider et al. [16]	Automated feature selection for emotion recognition in low-resource settings	7	41
Ozseven et al. [17]	Novel feature selection methods for SER	7	60.40
Latif et al. [18]	Transfer learning concept for enhancing SER	7	76.22
Proposed works	FAWT + OIFS + TELM	7	78.65
	Superlet + HHO + TELM	7	79.98
	Chirplet + CSA + TELM	7	80.63

**Table 14:** Performance comparison of classification accuracy—RAVDESS dataset.

Authors	Concept Used	Number of Classes	Classification Accuracy (%)
Jason and Kumar [19]	Machine learning techniques	8	80.21
Kwon [20]	Convolutional neural networks (CNNs)	8	79.50
Christy [21]	Multimodal SER using CNN	8	78.20
Masouri-Bensassi and Ye [22]	Spiking neural networks	8	83.60
Jalal et al. [23]	Capsule routing technique	8	77.02
Bhavan et al. [24]	Bagged SVM	8	75.69
Zeng et al. [25]	Spectrogram-based multi-task audio classification	8	64.48
Liu et al. [26]	Frequency cepstral coefficients with neural networks	8	79.80
Shegokar et al. [27]	Continuous wavelet transform (CWT)	8	60.10
Proposed works	FAWT + OIFS + TELM	8	84.19
	FAWT + HHO + TELM	8	85.76
	Chirplet + CSA + TELM	8	85.54

**Table 15:** Performance comparison of classification accuracy—SAVEE dataset.

Authors	Concept Used	Number of Classes	Classification Accuracy (%)
Vasuki and Aravindan [28]	A hierarchical classifier	7	83.78
Nguyen et al. [29]	Joint deep cross-domain transfer learning	7	69
Mekruksaranich et al. [30]	Negative emotion recognition	7	65.83
Hajarolasvadi and Demirel [31]	3D-CNN-based SER using K-means clustering and spectrograms	7	81.05
Tzinis et al. [32]	Integrated recurrence dynamics for SER	7	80.20
Sugan et al. [33]	Cepstral feature comparison for SER	7	78.60
Yogesh et al. [34]	Hybrid Particle Swarm Optimization (PSO)-based biogeography optimization	7	78.44
Proposed works	Chirplet + OIFS + TELM	7	83.94
	Chirplet + HHO + TELM	7	83.21
	KSTDIS + CSA + TELM	7	83.11

**Table 16:** Performance comparison of classification accuracy—Berlin Emo-DB dataset.

Authors	Concept Used	Number of Classes	Classification Accuracy (%)
Chen et al. [35]	A two-layer fuzzy multiple random forest concept	7	87.85
Daneshfar et al. [36]	Modified quantum-behaved PSO	7	82.82
Wang et al. [37]	Wavelet packet analysis	7	79.50
Guizzo et al. [38]	Multi-time-scale convolution	7	70.97
Zamil et al. [39]	Voting mechanisms	7	64.52
Alvarez et al. [40]	Stacked generalization method	7	82.45
Badshah et al. [41]	Divide-and-conquer-based ensemble technique	7	82.00
Proposed works	Superlet + OIFS + TELM	7	87.46
	KSTDIS + HHO + TELM	7	87.57
	KSTDIS + CSA + TELM	7	89.77

## Data Availability

The original data presented in this study are openly available in the EMOVO dataset [11], RAVDESS dataset [12], SAVEE dataset [13], and Berlin EMO-DB dataset [14].
